# Translation—Stomatitis

**Published:** 1891-06

**Authors:** Marie Pierre, M. V. del Valle

**Affiliations:** U. of M. Dental Class, 1891


### Translation—Stomatitis.

BY DR. MARIE PIERRE.

Translated from the French by M. V. del Valle, U. of M. Dental Class, 1891.

The diseases of the mouth are very frequent. Placed at the orifice of the digestive tract, the mouth takes its part in all the perturbations that affect the different parts of this canal, it is moreover subject to special indispositions coming from functions of its own. Its physiological role is the more important for it serves deglutition, respiration and the articulation of sounds. The saliva which is abundantly furnished by six glands, during mastication, three at each side (paroted, submaxillary and sublingual) convert the oral cavity in a sort of stomach, making the foods suffer a primary incomplete digestion, but that prepares and makes easier the assimilation that later will be carried on in the stomach and circulatory apparatus. The mouth is lastly the site of gustation and is liable as much as the fingers to get wounded in the performance of its functions.

In good state of health the lips are rosy and fresh, sweet and voluptuous, the gums hard, shiny and smooth, encircling the teeth in a frame of coral; the breath is inodoreous, but it is going too far to require with the poet from a healthy mouth, a sweet and perfumed breath, as the intoxicating emanations of a rose.

The health of the mouth is not only a question of æsthetics but more; of the good estate of this organ depend the good performance of the digestive tract, and very often of the general health. The modern science little caring for the poetry of the mouth, show it to us as a true nest of microbes, and these invincible organisms searching only for the occasion to attempt continually to our existance.

We are to study to-day simple stomatitis, *i. e.*, the numerous inflammations of this mucous membrane that lines the mouth with its rosy tissue. We shall leave aside the stomatitis produced by wounds and destroyed teeth, that which comes from certain eruptive and inflammatory diseases, as variole, scarlatina, typhoid fever, etc., and all secondary stomatitis. We will endeavor to take up the primitive stomatitis, which we will divide into two heads, stomatitis of external cause, and stomatitis of internal cause. Placed at the entrance of the digestive canal, and subjected to more or less irritant action of gases, vapors, alimentary substances, medicaments, and of all that is taken either to the stomach or lungs, it is natural that when these substances are introduced, regardless of their action, they will congest the mucous membranes of the mouth. Strong vinegar, pepper, alcohol will inflame the mouth, certain acids and certain salts will produce scars. The kermes and vitiated tartar in contact with the buccal mucous membranes produce, an injection of the mucous membranes, the color of which varies from dark red to the violet, and may provoke thrush.

Sugar itself may cause in certain persons painful stomatitis and aphthous eruptions. It is probable that sugar in this case is converted into lactic acid in coming in contact with the mucous membranes of the mouth. There are yet two more different kinds of stomatitis of external cause which are very common; the stomatitis of smokers and that of the glass workers.

Under the action of the hot smoke the smoker inhales, the mucous membranes of the mouth is the site of erythema, and very often we find it affected to a great extent.

The stomatitis of glass workers has for cause the practice of blowing. This kind of stomatitis is generally well localized. It can be noticed in the mucous membranes of the mouth of glass workers, two opal-like plates, situated in the inside of the cheek, about the middle of the face, they are called proffesional plates. In old working men about glass factories, these plates are no more opal-like, but they take the aspect of false membranes, wrinkled and having sometimes the size of a five francs coin.

All these different kinds of stomatitis of external cause are characterized by an exasperating pain, sensibility to heat and cold, the contact of food and movements of mastication. The mouth feels hot, dry and pasty-like, the breath is offensive, taste is bad, but it can be easily remedied by emollient gargles, mouth washes of sodium borate and the tablets and potions of chlorate of potash.

The stomatitis of internal cause are most always due to derangements of the digestive organs.

In individuals subject to constipation, a lack of system or a simple cold, is manifested in the mucous membranes of the mouth, which becomes red and tumefied, the mouth feels hot and dry, but with no pain. This indisposition yields readily to a mild regimen, milk, green vegetables, vichy water at meal times, laxatives and cooling refreshments.

The stomatitis of dentition, more frequent with very young infants, is accompanied with diarrhœ, vomits, etc. It is necessary not to neglect this kind of stomatitis, because the troubles about the digestive canal will be exagerated. It would be well to wash the mouth frequently with warm water, and apply the following liquid to the gums:

R. Glycerine, 15 grammes.
Potassium Bromide, 5 centrgrammes.
Cocaine Chlorhydrate, 10 centigrammes.

The stomatitis produced by eruption of the third molar or the wisdom tooth is particularly painful and persistent. It is distinguished by sharp neuralgic pains irradiating upon the side of the face and forming ulcerations of the tongue and gums. This kind of stomatitis is treated by large emollient poultices on the cheeks, but frequent it is necessary to take the molar out, and sometimes to do this, a portion of the alveolar ridge must be forced out with the knife.

Cold produces sometimes a stomatitis, said to be pultaceous in character, because of the whitish coating very frequently found covering the mucous membranes of the mouth and the tongue. The dryness of the mouth and constrained deglutition, common symptoms in all stomatitis are still more pronounced in this form, which can be readily cured by washes of peroxide of hydrogen.

The stomatitis diphtheria and the stomatitis ulcerative, the two last forms are easily noticed because the symptoms preceeding them are of such a character as to indicate a serious trouble, two or three days of intense fever, fatigue, etc. Both are caused by dampness, cold and bad nourishment. They are not of serious character, but they bring with them marked trouble in the digestive functions.

In the stomatitis diphtheria, so called because the mucous membrane of the mouth is covered by false membranes, similar in appearance to those found in diphtheria, the treatment consists in raising up with the forceps, these false membranes and touch the parts with boracic acid or the juice of a lemon.

The stomatitis ulcerosa is characterized by ulcerations which frequently only affect but one side of the mouth and with preference the left. The evolutions of the ulcerations are as follows; There is already a plague projecting out of violet color which does not take long to soften, now the pulpy and grayish surface detaches itself and leaves uncovered a ready, bleeding ulcer, with irregular edges. From the fifteenth to the seventeenth day the work of reparation begins and cicatrization takes place. It is more painful than serious, the cure is rapid, the application of chalk to the ulcers is many a time sufficient. It is necessary though to combat depression by giving the patient small doses of quinine.

It is then shown that stomatitis has nothing of a serious character, it is dependent upon local troubles or general causes, which is necessary to arrest to bring about a cure.

To much prudence cannot be exercised in the use of gargarisms and mouth washes; the alum and chlorate of potash should be used as little as possible as they are the principal agents of caries of the teeth. It is better to suffer from stomatitis, which a hygienic condition of the mouth can cure, than to injure the teeth so frail. It is non-scientific to endeavor to remedy a malady by producing some other trouble of worst character.

There were over thirty-one hundred students at the various colleges of dentistry of this country for the sessions of 1890–91.

